# Axl Promotes Zika Virus Entry and Modulates the Antiviral State of Human Sertoli Cells

**DOI:** 10.1128/mBio.01372-19

**Published:** 2019-07-16

**Authors:** Daniel P. Strange, Boonyanudh Jiyarom, Nima Pourhabibi Zarandi, Xuping Xie, Coleman Baker, Hooman Sadri-Ardekani, Pei-Yong Shi, Saguna Verma

**Affiliations:** a Department of Tropical Medicine, Medical Microbiology, and Pharmacology, John A. Burns School of Medicine, University of Hawaii at Manoa, Honolulu, Hawaii, USA; b Wake Forest Institute for Regenerative Medicine, Wake Forest School of Medicine, Winston-Salem, North Carolina, USA; c Department of Urology, Wake Forest School of Medicine, Winston-Salem, North Carolina, USA; d Department of Biochemistry and Molecular Biology, Sealy Center for Structural Biology and Molecular Biophysics, University of Texas Medical Branch, Galveston, Texas, USA; e Department of Microbiology and Immunology, Sealy Center for Structural Biology and Molecular Biophysics, University of Texas Medical Branch, Galveston, Texas, USA; Mailman School of Public Health, Columbia University

**Keywords:** Axl receptor tyrosine kinase, Sertoli cells, Zika virus, interferon signaling, testes, testicular organoids, virus entry

## Abstract

Zika virus (ZIKV) is unique among mosquito-borne flaviviruses in its ability to be sexually transmitted. Persistent ZIKV infection in the testes, which are immune privileged organs, long after peripheral clearance suggests involvement of immunosuppressive pathways; however, the underlying mechanisms remain undetermined. We recently demonstrated that ZIKV infects human Sertoli cells (SC), the major cell type of the seminiferous epithelium responsible for maintaining the immune privileged compartment of seminiferous tubules. Recent reports have identified the TAM (Tyro3, Axl, Mer) receptor tyrosine kinase Axl as an entry receptor and/or immune modulator for ZIKV in a cell type-specific manner. Interestingly, the seminiferous epithelium exhibits high basal expression of the Axl receptor where it is involved in clearance of apoptotic germ cells and immunosuppression. Here, we show that Axl was highly expressed in SC compared to Leydig cells (LC) that correlated with robust ZIKV infection of SC, but not LC. Further, neutralization of Axl receptor and its ligand Gas6 strongly attenuated virus entry in SC. However, inhibition of Axl kinase did not affect ZIKV entry but instead led to decreased protein levels of suppressor of cytokine signaling 1 (SOCS1) and SOCS3, increased expression of interferon-stimulated genes (ISGs), and reduced ZIKV replication. Similarly, treatment of multicellular human testicular organoids with an Axl kinase inhibitor attenuated ZIKV replication and increased ISG expression. Together, our data demonstrate that Axl promotes ZIKV entry and negatively regulates the antiviral state of SC to augment ZIKV infection of the testes and provides new insights into testis antiviral immunity and ZIKV persistence.

## Abstract (precis)

Recent Zika virus (ZIKV) outbreaks have identified sexual transmission as a new route of disease spread not reported for other flaviviruses. ZIKV crosses the blood-testis barrier and establishes infection in seminiferous tubules, the site for spermatozoa development. Currently, there are no therapies to treat ZIKV infection, and the immune mechanisms underlying testicular persistence are unclear. We found that multiple human testicular cell types, except Leydig cells, support ZIKV infection. Axl receptor, which plays a pivotal role in maintaining the immunosuppressive milieu of the testis, is highly expressed in Sertoli cells and augments ZIKV infection by promoting virus entry and negatively regulating the antiviral state. By using testicular organoids, we further describe the antiviral role of Axl inhibition. The significance of our research lies in defining cross talk between Axl and type I interferon signaling as an essential mechanism of immune control that can inform therapeutic efforts to clear ZIKV from the testis.

## INTRODUCTION

Zika virus (ZIKV) is a mosquito-borne flavivirus of the *Flaviviridae* family. Following the 2015–2016 widespread epidemic in the Americas, ZIKV emerged as a teratogenic and sexually transmissible virus (1, 2), findings not reported for other flaviviruses. Detection of ZIKV in human sperm and semen for months after viremia has cleared (3–5) strongly suggests that the testes are the only organs in adult immunocompetent humans where ZIKV can establish persistence and thus presents a risk for pregnant women in areas where ZIKV is endemic and areas where ZIKV is not endemic to acquire ZIKV from male partners. To establish persistent infection in the testes, ZIKV must remain undetected from peripheral immunity and maintain low-level replication without causing severe cell death and inflammation. Although some of the potential cellular targets for ZIKV infection in the human testes have been identified (6–9), the mechanisms by which ZIKV infects these cells and establishes testicular persistence remain undefined.

The testis immune environment is inherently immunosuppressive and tightly regulated by a communication network between resident cells of the interstitial space (testosterone-producing Leydig cells [LC] and testicular macrophages) and seminiferous tubules (Sertoli cells [SC], peritubular myoid cells [PMC], and spermatogonial stem cells [SSC]) through the production of hormones, cytokines, and chemokines (10). SC are large columnar-type cells that, in addition to forming the blood-testis barrier and supporting spermatogenesis (11), also induce innate immune response to pathogens, including viruses by producing cytokines and interferons (IFNs) (12–16). To maintain the local immunosuppressive milieu of the testis, SC express TAM (Tyro3, Axl, Mer) receptor tyrosine kinases, notably Axl, that mediate clearance of apoptotic germ cells (autoantigens) when bound to their ligands, growth arrest-specific 6 (Gas6) and/or protein S1 (15, 17, 18). TAM receptors play a pivotal role in dampening innate antiviral response by two defined mechanisms. First, TAM receptors colocalize with the alpha/beta interferon (IFN-α/β) receptor subunit 1 (IFNAR1) to form a TAM-IFNAR complex that effectively subverts and redirects canonical IFNAR function (19). Second, this TAM-IFNAR interaction, in concert with TAM ligand binding, leads to phosphorylation of STAT1 (signal transducer and activator of transcription 1) homodimers that translocate to the nucleus and initiate the expression of suppressor of cytokine signaling 1 (SOCS1) and SOCS3 (19). SOCS1 and SOCS3 proteins further suppress type I IFN production by inhibiting Janus kinases (JAKs) and Toll-like receptor (TLR) signaling (19).

Recent reports have identified Axl as a potential entry factor for ZIKV and other flaviviruses (20–22), which is thought to be facilitated by sequestration of Axl ligands (e.g., Gas6) to exposed phosphatidylserine on the ZIKV envelope membrane (20, 22), a process termed as viral apoptotic mimicry (23). However, data on the role of Axl in ZIKV entry is conflicting and appears to be cell type specific (20, 21, 24). While Axl is required for efficient infection of human endothelial cells, skin cells, astrocytes, and brain glial cells (20, 21, 24–26), it is reportedly dispensable for ZIKV infection of human neural progenitor cells (hNPC) and cerebral organoids (27). Axl also appears to be dispensable for ZIKV infection in mice lacking normal IFNAR function (28, 29); however, the absence of type I IFN signaling in this model may partially explain the discrepancies between mouse *in vivo* and human *in vitro* studies. Interestingly, according to the Human Protein Atlas, the testis is one of two human tissues in which Axl has been shown to be highly expressed (30).

We, as well as others, have shown that human SC are highly susceptible to prolonged ZIKV infection without cytopathic effect despite the induction of antiviral response, including production of type I IFN (6, 7, 12), suggesting that SC may be a preferred target for ZIKV infection and persistence in the testes. Although a recent study reported that blocking Axl receptor alone, and not other TAM receptors, resulted in attenuated ZIKV replication in human SC (7), the specific role of Axl in ZIKV infection of the testes is yet to be characterized. Together, these observations and the known immunosuppressive role of Axl led us to hypothesize that constitutive expression of Axl enhances entry of ZIKV and suppresses antiviral response in human SC to promote robust and persistent infection. We report here that ZIKV can infect human SC, PMC, and SSC. Conversely, we show that primary human LC are resistant to ZIKV infection, which correlates with lower Axl expression compared to SC. We also demonstrate the dual role of Axl in augmenting ZIKV infection of SC, by promoting ZIKV entry and suppressing the antiviral state. Further, we demonstrate the antiviral role of Axl kinase inhibitor R428 by its ability to attenuate productive ZIKV infection of SC and multicellular human testicular organoids (HTO). Our data collectively provide new insights into ZIKV persistence and antiviral defense in the testis, a relatively understudied immune privileged organ.

## RESULTS

### ZIKV can infect multiple testicular cell types.

**FIG 1:**
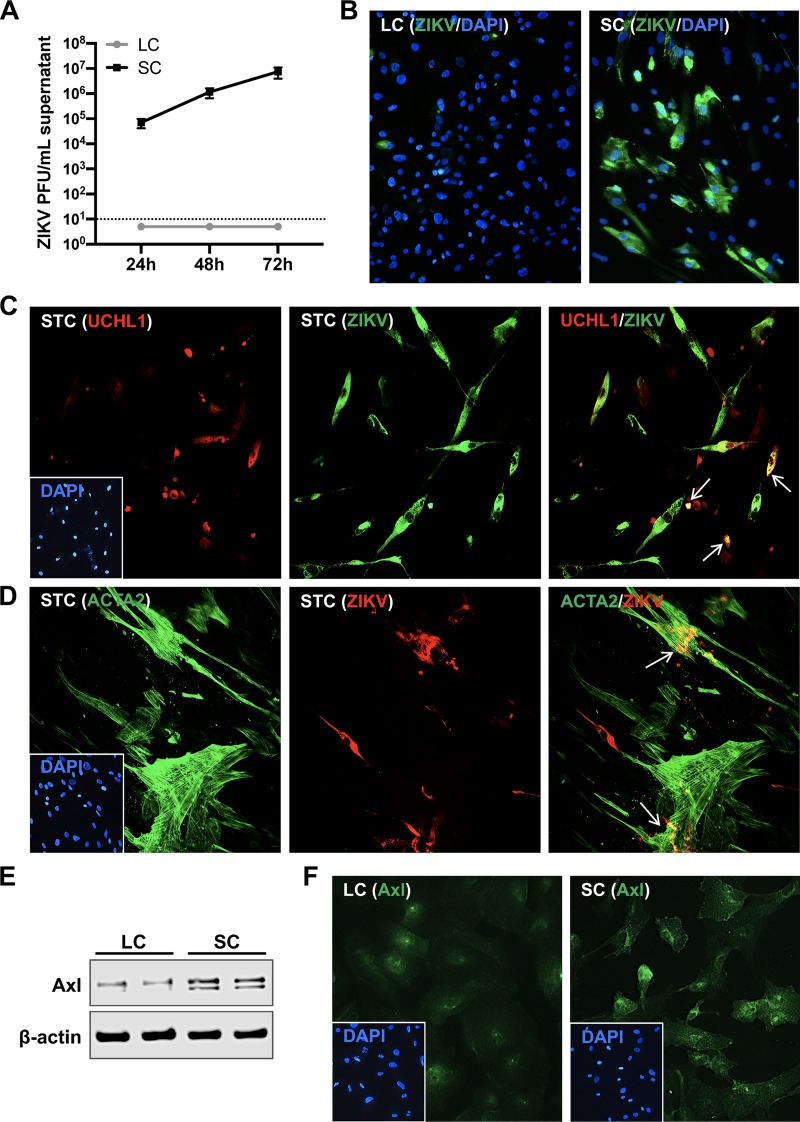
ZIKV infects multiple testicular cell types. (A) SC and LC were infected at an MOI of 1, and progeny virus produced was determined by plaque assay at 24, 48, and 72 h postinfection. Data represent averages of ≥3 independent experiments; the dotted line indicates the minimal detection limit of the assay. (B) Representative image of ZIKV immunofluorescence staining in mock-infected and infected LC and SC at 48 h postinfection. Cells were stained for ZIKV using antibody against the envelope protein (anti-ZIKV-E [green]) and nuclei stained with 4′,6′-diamidino-2-phenylindole (DAPI) (blue). Mixed STC were infected with ZIKV at an MOI of 1 and stained for DAPI, SSC marker (anti-UCHL1 [red]) (C), PMC marker (anti-ACTA2 [green]), and ZIKV-E (green for SSC and red for PMC) (D) at 48 h postinfection. White arrows in merged images indicate colocalization of cell markers with ZIKV-E. (E) Whole-cell extracts from uninfected SC and LC were subjected to Western blotting and stained for Axl and β-actin; each lane shows the results for an independent experiment for each cell type. (F) Representative images of Axl immunofluorescence staining (anti-Axl [green]) in uninfected LC and SC.

To compare the relative infectivity of different testicular cell types, we first examined ZIKV infection in primary human Sertoli cells (SC), Leydig cells (LC), and mixed seminiferous tubule cells (STC) containing SC, peritubular myoid cells (PMC), and spermatogonial stem cells (SSC). We did not detect any plaques in the supernatant from infected LC at any time point compared to SC (Fig. 1A). LC were also infected with higher multiplicities of infection (MOIs) of 5 and 10; however, no infectious virions were detected in the supernatant (data not shown). Immunofluorescence assay (IFA) also confirmed robust ZIKV infection of SC, whereas little immunoreactivity to ZIKV antigen was detected in LC (Fig. 1B). ZIKV infection in mixed STC cultures was confirmed by immunofluorescence staining of ZIKV envelope (E) protein at 48 h postinfection. As shown in Fig. 1C, SSC stained with undifferentiated spermatogonia marker UCHL1 were also positive for ZIKV-E antigen (white arrows, merged image). Similarly, PMC stained with ACTA2 also exhibited immunoreactivity to ZIKV-E antigen (white arrows, merged image in Fig. 1D). To determine the correlation between ZIKV infectivity and Axl expression, we measured the protein levels and surface expression of Axl in SC and LC by Western blotting and IFA, respectively. Immunoblotting demonstrated substantially lower levels of Axl protein in LC (Fig. 1E) that also correlated with diminished cell surface staining of Axl in LC compared to SC (Fig. 1F). Since SSC cannot be cultured alone without feeder SC (31), ZIKV infection between these cells and other testicular cells could not be compared. Collectively, these data indicate that multiple cell types of the seminiferous tubule compartment can support infection, whereas LC, an important cell type of the interstitial space, are resistant to ZIKV infection, and that the differences in Axl protein expression may partially explain the disparity in ZIKV infectivity of SC and LC.

### Axl augments ZIKV infection of human SC.

**FIG 2:**
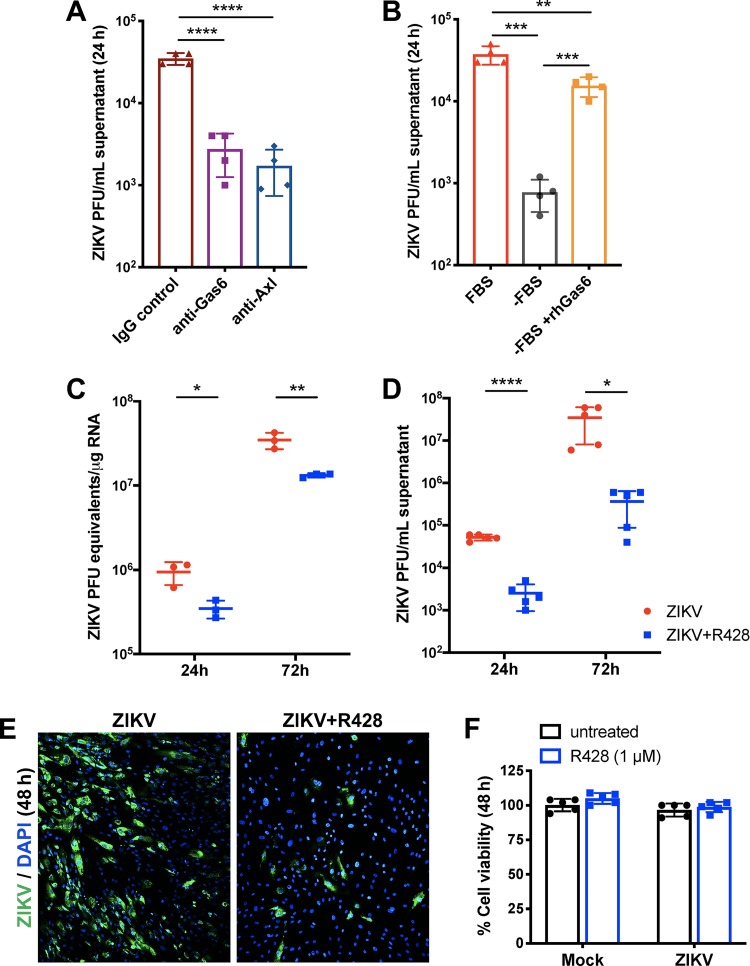
Axl augments ZIKV infection of human SC. (A) ZIKV progeny titers measured in the supernatant of SC treated with anti-Gas6 neutralizing antibody (50 μg/ml) or anti-Axl blocking antibody (10 μg/ml) or goat IgG control (50 μg/ml) by plaque assay at 24 h postinfection. (B) ZIKV progeny titers were also determined 24 h postinfection in SC grown in the presence of serum (FBS) or absence of serum (−FBS) and compared to serum-deprived SC treated with rhGas6 (5 nM) upon infection (−FBS +rhGas6). (C) ZIKV RNA copies measured in infected SC in the presence and absence of Axl kinase inhibitor R428 (1 μM) by qRT-PCR and expressed as ZIKV PFU equivalents per microgram of RNA. (D) ZIKV progeny titers measured by plaque assay at 24 h and 72 h postinfection of SC treated with R428. (E) ZIKV infection of SC treated with R428 (1 μM) was also determined by IFA via staining of ZIKV (anti-ZIKV-E [green]) at 48 h postinfection. (F) Cell viability was evaluated following R428 treatment (1 μM) in mock-infected and infected SC by CellTiter 96 AQueous One Solution at 48 h postinfection, and the percent cell viability was calculated by comparison to the value for mock-infected cells at the corresponding time point. DMSO vehicle was used as untreated control for R428 experiments. Values are averages ± standard deviations (SD) (error bars) from 3 to 5 independent experiments with cells infected at an MOI of 1. Values that are significantly different are indicated by a bar and asterisks as follows: *, *P* < 0.05; **, *P* < 0.01; ***, *P* < 0.001; ****, *P* < 0.0001.

To determine whether high Axl expression promoted ZIKV infection, SC were preincubated with an anti-Axl antibody that blocks Axl receptor ligand binding, and infectious ZIKV progeny was subsequently quantified in the supernatant 24 h postinfection. As seen in Fig. 2A, ZIKV infection was significantly attenuated by anti-Axl antibody compared to infected SC treated with an IgG control. To further confirm the role of Gas6 in mediating virus infection, we also measured virus replication in SC pretreated with an anti-Gas6 antibody specific for the Axl binding domain of Gas6. Consistent with anti-Axl treatment, we found that ZIKV infection was reduced by more than 90% in cultures treated with anti-Gas6 compared to the IgG control (Fig. 2A). Since the source of Gas6 is mostly serum present in the culture media, we further assessed ZIKV infection in serum-deprived SC. As seen in Fig. 2B, the absence of serum significantly reduced ZIKV titers by more than 95%, and this phenomenon was markedly restored by the addition of exogenous recombinant human Gas6 (rhGas6).

To next evaluate whether downstream Axl kinase activity also has any effect on ZIKV infection, we infected SC in the presence of R428, a selective small molecule inhibitor of Axl kinase activity (32). We observed almost a 50% reduction in ZIKV RNA at both 24 and 72 h postinfection (Fig. 2C). Consistent with our Axl receptor blockade results, the levels of mature infectious ZIKV in the supernatant were reduced by more than 1 log unit (∼90%) at both time points in R428-treated SC (Fig. 2D). Reduction of ZIKV infection in R428-treated SC was also confirmed by IFA 48 h postinfection (Fig. 2E). We further validated that reduced virus replication in R428-treated SC was not due to compromised cell viability from potential toxicity effects of R428 (Fig. 2F). Taken together, these results indicate that Axl-Gas6 engagement and downstream Axl kinase activity are required for robust ZIKV infection of human SC.

### Axl receptor binding promotes ZIKV entry of human SC.

**FIG 3:**
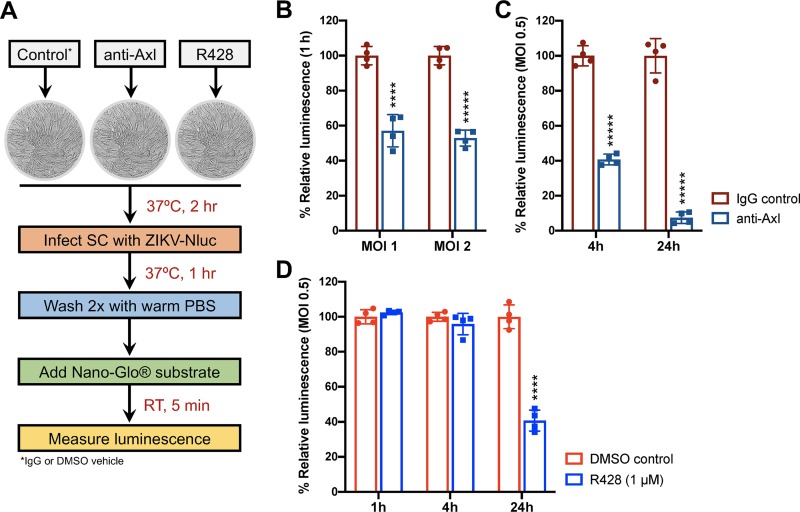
Axl receptor binding promotes ZIKV entry of human SC. (A) Schematic of experimental design for evaluating ZIKV-Nluc entry of SC using Nano-Glo luciferase assay system. (B) Measurement of luciferase activity (luminescence) in SC at 1 h postinfection with ZIKV-Nluc at an MOI of 1 and an MOI of 2 following Axl receptor blockade using anti-Axl antibody (10 μg/ml). (C) ZIKV-Nluc replication in SC was also determined at 4 h and 24 h postinfection with an MOI of 0.5 following Axl receptor blockade. (D) SC were treated with selective Axl kinase inhibitor R428 (1 μM), and ZIKV-Nluc replication was determined by measuring luminescence at 1 h, 4 h, and 24 h postinfection with an MOI of 0.5. The average values for matched time point controls were used as the baseline to determine the percent change in luminescence. Goat IgG and DMSO vehicle were used as controls for anti-Axl and R428 treatment assays, respectively. Values are means ± SD (error bars) from at least four independent infections. ****, *P* < 0.0001; *****, *P* < 0.00001.

Since Axl is proposed to be a ZIKV entry receptor as well as immune modulator in recent studies, it was important to delineate the specific entry versus immune modulatory role of Axl in SC. Our results using anti-Gas6 and serum-deprived media (Fig. 2) also suggested that Axl is potentially involved in ZIKV entry of human SC. To confirm and investigate this phenomenon further, we performed a ZIKV entry assay using a ZIKV-reporter encoding *Nano* luciferase (ZIKV-Nluc). Since ZIKV is a positive-sense RNA virus, the Nluc gene is expressed immediately upon entry of the ZIKV-Nluc genome into the host cytoplasm. Therefore, the detection of Nluc activity at early time points (1 and 4 h) is considered a measure of virus entry, whereas Nluc activity at 24 h represents the level of replicating virus. SC were preincubated with anti-Axl, R428, or respective controls (IgG or dimethyl sulfoxide [DMSO] vehicle) prior to infection with ZIKV-Nluc and subsequently measured for Nluc activity (luminescence) postinfection (Fig. 3A). We observed nearly a 50% reduction in Nluc activity in SC treated with anti-Axl compared to IgG control by 1 h postinfection with an MOI of 1 and an MOI of 2 (Fig. 3B). Further, we observed a >60% and >90% reduction in Nluc activity in SC infected with an MOI of 0.5 by 4 h and 24 h postinfection, respectively (Fig. 3C). In contrast, treatment of SC with R428 had no effect on Nluc activity at early time points, but activity was reduced by 60% at 24 h postinfection with an MOI of 0.5 (Fig. 3D). These results collectively indicate that Axl receptor binding, not Axl kinase signaling, promotes ZIKV entry of human SC.

### Axl suppresses the antiviral state of human SC to promote ZIKV infection.

**FIG 4:**
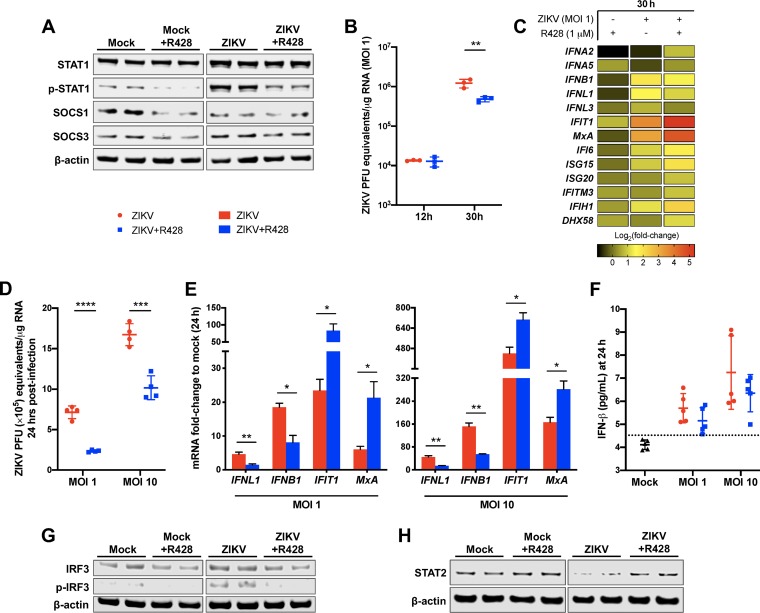
Axl suppresses the antiviral state of human SC. (A) Protein expression of total and phosphorylated STAT1 (p-STAT1), SOCS1, and SOCS3 was determined in total cell lysates from mock-infected and ZIKV-infected (MOI of 10) SC following R428 treatment (1 μM) by Western blotting. β-Actin was used as a housekeeping loading control. Each lane shows the results from an independent experiment. SC with or without R428 treatment (1 μM) were infected with ZIKV at an MOI of 1 and at 30 h postinfection. (B) ZIKV RNA copies were determined by qRT-PCR and expressed as ZIKV PFU equivalents per microgram of RNA. (C) mRNA fold change of ISGs (*IFIT1*, *MxA*, *IFI6*, *ISG15*, *ISG20*, *IFITM3*, *IFIH1*, and *DHX58*), type I IFN genes (*IFNA2*, *IFNA5*, and *IFNB1*), and type III IFN genes (*IFNL1* and *IFNL3*) compared to mock-infected cells were measured using qRT-PCR by normalizing to *GAPDH*. (D) ZIKV RNA copies were also determined in SC following R428 treatment (1 μM) and infection with an MOI of 1 and an MOI of 10 at 24 h. (E) The effect of infectious dose on the mRNA expression of *IFIT1*, *MxA*, *IFNL1*, and *IFNB1* was determined in SC with and without R428 treatment by normalizing to *GAPDH* (values are means ± standard errors of the means [SEM] [error bars] from three independent experiments). (F) Secreted IFN-β protein levels in SC supernatant at 24 h postinfection was determined by ELISA. The dotted line indicates the minimal detection limit of the assay. (G and H) The effect of R428 treatment (1 μM) on total and phosphorylated IRF3 (p-IRF3) protein expression (G) and total STAT2 protein expression (H) was determined in both mock-infected and infected SC at 24 h postinfection (MOI of 10) by Western blotting. DMSO vehicle was used as untreated control for all assays. *, *P* < 0.05; **, *P* < 0.01; ***, *P* < 0.001; ****, *P* < 0.0001.

Axl kinase is known to bind to and coopt IFNAR1 to effectively subvert canonical IFNAR function toward the immunosuppressive Axl-IFNAR signaling axis, which leads to STAT1 homodimer phosphorylation and subsequent induction of SOCS1 and SOCS3 (19). Therefore, to investigate whether Axl kinase augments ZIKV infection of human SC by negatively regulating antiviral response, we first evaluated the effect of R428 treatment on protein levels of STAT1/p-STAT1 (phosphorylated STAT1), SOCS1, and SOCS3. We found that ZIKV infection (MOI of 10) induced phosphorylation of STAT1 and that R428 treatment reduced levels of p-STAT1 in both mock-infected and infected SC (Fig. 4A). Similarly, levels of SOCS1 and SOCS3 were diminished in R428-treated SC compared to untreated controls, suggesting that R428 disrupts the Axl-IFNAR signaling cascade.

To further characterize the downstream consequences, we next compared the expression of type I IFN (*IFNA2*, *IFNA5*, and *IFNB1*) and type III IFN (*IFNL1* and *IFNL3*) genes and a panel of key interferon-stimulated genes (ISGs) in R428-treated versus untreated SC at 30 h postinfection (MOI of 1). Importantly, we observed no difference in intracellular ZIKV RNA between R428-treated and untreated SC by 12 h postinfection (Fig. 4B), again demonstrating that R428 treatment does not hinder ZIKV entry of human SC. Consistent with our previous observations, by 30 h postinfection, ZIKV RNA was reduced in R428-treated SC compared to untreated SC (Fig. 4B), which correlated with a modest decrease in the transcript levels of *IFNB1* and *IFNL1* in R428-treated SC compared to untreated control. However, despite the decreased mRNA levels of IFNs in R428-treated SC, we observed induction of many ISGs, including *IFIT1*, *ISG15*, *IFI6*, *IFIH1*, and the IFN-dependent ISG *MxA* compared to untreated SC (Fig. 4C).

We next questioned whether these modulated antiviral effects were attributable and/or sensitive to infectious dose. Thus, we evaluated *IFIT1*, *MxA*, *IFNB1*, and *IFNL1* expression in R428-treated SC following ZIKV infection at an MOI of 1 and an MOI of 10. At 24 h postinfection, 60% and 40% reduction in ZIKV RNA was observed in R428-treated SC infected at an MOI of 1 and an MOI of 10, respectively, compared to untreated SC (Fig. 4D) that correlated with reduced transcripts of *IFNL1* and *IFNB1* (Fig. 4E). These differences were further validated by ELISA, which demonstrated a similar trend of reduced IFN-β levels in the supernatant of R428-treated SC compared to untreated SC (Fig. 4F). However, in spite of reduced IFN, we again observed higher expression of *IFIT1* and *MxA* in R428-treated SC at 24 h postinfection with an MOI of 1 and an MOI of 10 (Fig. 4E). Since IFN regulatory transcription factor 3 (IRF3) is a master regulator of IFN type I (IFN-I) production downstream of viral RNA-sensing pattern recognition receptors (PRRs) (33), we also assessed the activation status of this transcription factor. We found that although ZIKV infection induced phosphorylation of IRF3 in SC, this was dramatically diminished by R428 treatment (Fig. 4G), suggesting that *IFNB1* and *IFNL1* transcription in SC is responsive to ZIKV genome replication and not directly modulated by Axl-IFNAR signaling. Furthermore, it is well documented that ZIKV NS5 mediates degradation of human STAT2 (34, 35), a component of the ISGF3 transcription factor complex that promotes the expression of various ISGs downstream of activated type I and III IFN receptors (36, 37). In agreement, we found that STAT2 protein levels were reduced in ZIKV-infected SC; however, this was mitigated in R428-treated SC (Fig. 4H), most likely due to reduced ZIKV replication. Taken together, these data suggest that R428 interferes with the canonical interaction of Axl and IFNAR1 in human SC to potentiate IFNAR-dependent antiviral state during ZIKV infection.

### Axl kinase augments ZIKV infection of HTO.

**FIG 5:**
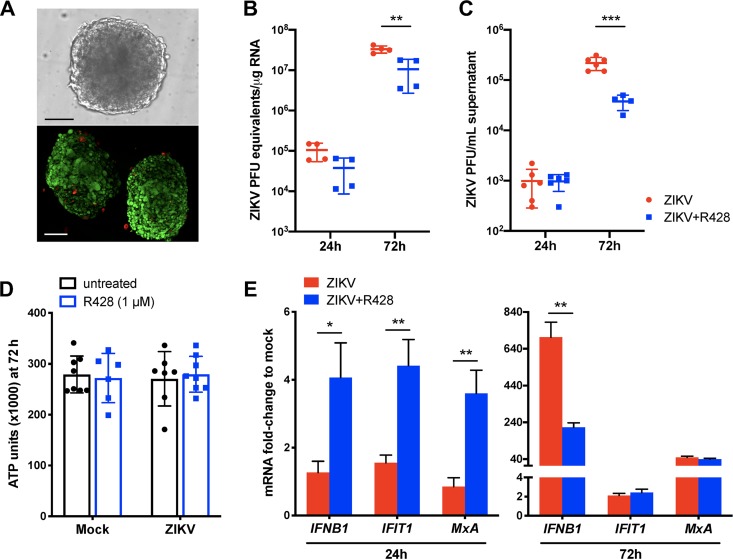
Axl kinase augments ZIKV infection of HTO. (A) Morphology and viability evaluation of HTO. Bright-field imaging (top) and LIVE/DEAD Confocal 3D Z-Stack projection (bottom) of representative organoids after post-thaw recovery in culture (red fluorescence depicts dead cells and green depicts live cells). Bars, 100 μm. (B and C) HTO were infected with ZIKV in the presence or absence of R428 (1 μM) and at 24 h and 72 h postinfection, ZIKV RNA copies (B) and progeny titers in the supernatant (C) were determined using qRT-PCR (30 HTO per data point) and plaque assay (10 HTO per data point), respectively. (D) Viability of R428-treated and untreated HTO was determined using CellTiter Glo 3D Cell Viability Assay at 72 h postinfection (values are averages ± SD [error bars] from ≥6 HTO per group). (E) Expression of *IFNB1*, *IFIT1*, and *MxA* transcripts was determined using qRT-PCR and expressed as mRNA fold change to mock-infected cells by normalizing to *GAPDH* (values are means ± SEM [error bars} of four data points, and each data point is a pool of RNA from 30 HTO). HTO were infected with 10^5^ PFU, and DMSO vehicle was used as the untreated control for all assays. *, *P* < 0.05; **, *P* < 0.01; ***, *P* < 0.001.

We recently demonstrated that human testicular organoids (HTO), composed of four different cell types (SC, LC, PMC, and SSC), are highly permissible to ZIKV infection (38). These HTO produce testosterone continuously and can partially support germ cell differentiation and thus recapitulate the cell type diversity and function of the testes better than any other existing *in vitro* system (39). Therefore, to further validate the role of Axl in ZIKV infection of a multi-testicular cell model, HTO (Fig. 5A) were infected with ZIKV in the presence and absence of R428, and virus titers and host antiviral response were evaluated. As shown in Fig. 5B and C, no significant decrease in ZIKV titers was detected at the early time point postinfection (24 h); however, by 72 h postinfection, both ZIKV RNA and ZIKV infectious progeny in the supernatant were significantly diminished in R428-treated HTO compared to untreated HTO. Importantly, no change in ATP production (as a marker of HTO viability) was observed between R428-treated and untreated HTO regardless of ZIKV infection (Fig. 5D), indicating that the differences in ZIKV titers were not due to compromised HTO survival. Host response analysis demonstrated increased *IFNB1*, *IFIT1*, and *MxA* expression in R428-treated HTO at 24 h postinfection (Fig. 5E). By 72 h postinfection, *IFNB1* was reduced in R428-treated compared to untreated HTO (Fig. 5E) and thus correlated with decreased ZIKV titers. However, unlike SC alone, expression of *IFIT1* and *MxA* was similar between both groups at 72 h postinfection (Fig. 5E), most likely due to the presence of other cell types in HTO with different infectivity profiles. Nonetheless, these results support our overall hypothesis that Axl promotes ZIKV infection in the testes.

### The antiviral state of LC is elevated compared to SC under basal conditions.

**FIG 6:**
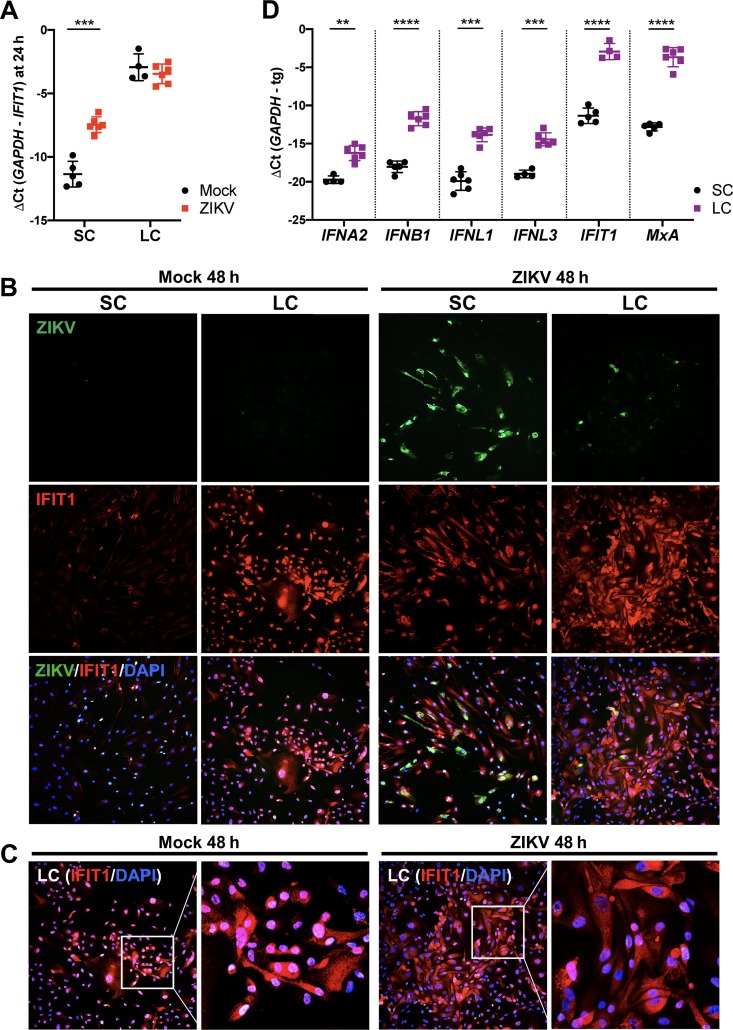
The antiviral state of LC is elevated compared to that of SC under basal conditions. (A) IFIT1 mRNA expression in mock-infected and ZIKV-infected SC and LC reported as a change in cycle threshold (ΔCt) between *GAPDH* and *IFIT1* transcripts 24 h after ZIKV infection (MOI of 1). Values are means ± SD (error bars) for 4 to 6 independent experiments. (B) The effect of ZIKV infection on IFIT1 protein expression was evaluated in SC and LC at 48 h postinfection (MOI of 1) by staining for ZIKV (anti-ZIKV-E [green]) and IFIT1 (anti-IFIT1 [red]). (C) IFIT1 protein localization following infection was evaluated by merging nucleus staining (DAPI [blue]) with IFIT1 staining (anti-IFIT1 [red]). Nuclear localization of IFIT1 is indicated by overlapping blue and red staining (magenta). (D) mRNA expression of antiviral genes—type I IFNs (*IFNA2* and *IFNB1*), type III IFNs (*IFNL1* and *IFNL3*), and *IFIT1* and *MxA*—was determined and compared between naive SC and LC using qRT-PCR and reported as ΔCt between *GAPDH* and target gene (tg) transcripts. **, *P* < 0.01; ***, *P* < 0.001; ****, *P* < 0.0001.

Our data depicted in Fig. 4 suggest that *IFIT1* and *MxA* expression may serve as a marker of the antiviral state and restricted ZIKV infection. *IFIT1*, an antiviral gene highly induced by ZIKV (8, 12), is shown to be expressed in both an IFN-dependent and -independent manner (37, 40). Interestingly, while IFITs generally are not expressed in cells under basal conditions (40), Human Protein Atlas (HPA) immunohistochemistry data demonstrate elevated basal expression of IFIT1 in the testis interstitial compartment (30) consisting mostly of LC. Thus, we hypothesized that differences in the basal antiviral state, as indicated by *IFIT1* expression, may partially account for the differential susceptibility of LC and SC to ZIKV infection. To probe this question further, we evaluated mRNA levels of IFIT1 in SC and LC following ZIKV infection. Consistent with HPA data, we found that naive LC exhibited significantly higher levels of IFIT1 mRNA compared to naive SC (Fig. 6A). Further, at 24 h postinfection, *IFIT1* transcription was upregulated in SC yet failed to reach levels comparable to those observed in LC (Fig. 6A). Consistent with mRNA data, immunofluorescence staining detected markedly higher levels of IFIT1 protein in mock-infected and infected LC compared to SC, despite the detection of robust ZIKV infection in SC (Fig. 6B). Furthermore, also consistent with mRNA data at 24 h, IFIT1 protein was induced in SC by ZIKV infection at 48 h, yet the immunoreactivity of IFIT1 in infected SC was still much lower compared to LC (Fig. 6B). Interestingly, we observed nuclear localization of IFIT1 in mock-infected LC that was noticeably more distributed to the cytoplasm following ZIKV infection (Fig. 6C). We next questioned whether *IFIT1* was the only antiviral gene with higher basal expression in LC than in SC. Comparison of the mRNA levels of type I and III IFNs and MxA in naive LC and SC demonstrated that the basal expression of all genes was significantly higher in LC than in SC (Fig. 6D). Overall, these results provide additional insights into the response of LC to ZIKV infection; however, further investigation is warranted to carefully determine whether heightened basal expression of antiviral genes, including *IFIT1*, does indeed contribute to the resistance of LC to ZIKV infection.

## DISCUSSION

Detection of ZIKV in human semen and sperm for a long period after acute infection highlights the testis as a site for virus persistence in immunocompetent individuals.

Our previous finding that, in spite of induction of key antiviral pathways, ZIKV can still establish robust and long-term infection in SC led to another important question—are there any specific mechanisms that allow these immune suppressive cells to become the target of persistent ZIKV infection in the testes? The data in the present study highlight the following results. (i) Multiple cells present in the seminiferous tubules support ZIKV infection, whereas LC, an important cell type of the interstitial space, do not. (ii) Axl is highly expressed in SC and augments ZIKV infection by promoting virus entry and negatively regulating the antiviral state. (iii) Inhibition of Axl kinase attenuates ZIKV infection of multicellular HTO. (iv) Resistance of LC to ZIKV can be potentially attributed to lower Axl surface expression and elevated antiviral state under basal conditions compared to SC.

Susceptibility to ZIKV infection is shown to be highly cell type specific, and here we show that ZIKV can infect all cell types of seminiferous tubules, with SC clearly supporting more robust infection. The detection of ZIKV in SSC and PMC (Fig. 1) is in agreement with a recent study by Matusali and colleagues (8) who reported virus replication in these cell types in human testis explants; however, they did not detect strong replication in SC (8). Nonetheless, our findings further validate the notion that direct ZIKV infection of germ cells may impair the spermatogenesis process and lead to altered sperm parameters observed in infected human males (41). Interestingly, we found that LC were resistant to ZIKV infection. While IFNAR-deficient mouse studies demonstrated the presence of ZIKV and induction of inflammation in LC (42), we did not detect ZIKV replication in LC even when the cells were infected at an MOI of 10. Our results however, agree with Kumar and colleagues who reported ZIKV infection in human SC, but not in LC (7).

Cell signaling molecules associated with the suppression of immune response, including Axl, are highly expressed in the mammalian testes, predominantly in seminiferous tubules (15, 18, 43, 44). Axl is the only TAM receptor shown to play a significant role in augmenting ZIKV infection in a cell type-specific manner (20, 21, 24–27). Our data reveal a dual role of Axl in augmenting ZIKV infection of human SC. It was only through Axl receptor blockade, and not inhibition of Axl kinase, that led to significant reduction in ZIKV entry of SC, suggesting that Axl-mediated entry does not require Axl kinase activity, an observation consistent with a previous study in human glial cells (20). Although Kumar and colleagues (7) reported an antiviral effect of blocking Axl in human SC, this study did not delineate the immunomodulatory role of Axl. Using a ZIKV-luciferase reporter assay system (45), we were able to further dissect the specificity of Axl receptor engagement in promoting ZIKV entry and Axl kinase activity in modulating the antiviral state of human SC.

The primary function of Axl in SC, as determined by rodent studies, is to mediate phagocytosis of apoptotic germ cells and to maintain immune homeostasis by negatively regulating innate immunity (15, 18). In human cell types, Axl is also shown to dampen IFNAR signaling and TLR-mediated inflammation (24, 46); however, the ability of Axl to restrict the antiviral response in the human testis is not yet clearly understood. We and others have shown that human SC are capable of producing type I IFN and key ISGs following infection with ZIKV (6, 7, 12). We further show here that ZIKV infection of SC induces expression and phosphorylation of STAT1, an important molecule involved in IFNAR and Axl-IFNAR signaling (19). Dramatic reduction in p-STAT1 levels corresponded with decreased levels of SOCS1 and SOCS3 in both R428-treated mock-infected and infected SC. These findings follow a similar pattern of reduced SOCS1 observed in Axl-deficient astrocytes following ZIKV infection (24). More importantly, inhibition of Axl kinase further enhanced ZIKV-induced upregulation of ISGs, particularly *MxA* and *IFIT1*. *MxA* is an IFN-dependent ISG induced downstream of IFNAR activation (36, 37), whereas *IFIT1* is induced both by IFN and directly by IRF3 activation through viral RNA-sensing pathways (e.g., RIG-I and TLR3) (37, 40). However, the strong induction of *IFIT1* in spite of reduced IRF3 phosphorylation observed in R428-treated SC suggests that the further boost in *IFIT1* expression is a result of Axl kinase inhibition. The similar effect of increased expression of ISGs in R428-treated HTO, although not as dramatic as SC alone and probably due to the presence of other cell types, including LC, further validates the antiviral role of Axl in a multi-testicular-cell model. Collectively, these findings suggest that Axl kinase inhibition in SC disrupts the Axl-IFNAR1 complex, which in turn liberates canonical IFNAR function to allow increased expression of IFN-dependent ISGs (e.g., *IFIT1* and *MxA*) in both infected and neighboring uninfected cells to ultimately restrict virus replication.

Another interesting observation of our data was the association between resistance of LC to ZIKV and basal IFIT1 expression. Although our data agree with Human Protein Atlas data demonstrating high immunoreactivity to IFIT1 in LC (30), the specific function of IFIT1 has not yet been characterized in these cells. IFIT1 primarily localizes to the cytoplasm, where it implements its effector function by antagonizing viral RNA translation (40). A recent report demonstrated that IFIT1 can also localize to the nucleus, where it was shown to serve as a positive regulator for p-IRF3 recruitment at the *IFNB1* and ISG loci (47). Interestingly, although IFIT1 levels did not further increase following ZIKV infection, immunostaining detected apparent nuclear localization of IFIT1 in addition to diffused staining in the cytoplasm of infected LC. Thus, we speculate that the high basal levels of IFIT1 in the nuclei of LC may prime these cells to fight virus infection by indirectly promoting immediate antiviral response. However, the exact mechanistic role of nuclear localization of IFIT1 in LC and its effects on virus infection require further investigation. These data together with our observation of low overall Axl expression and heightened basal antiviral gene expression in human LC may partially explain why these cells do not support ZIKV infection. However, this finding does not agree with mouse studies that reported ZIKV infection in mouse LC stem cells (42) and thus further confirms the differences between human and mouse testis physiology.

Our understanding of testis immunity to viruses is currently very limited. With no specific treatment for clearing testicular ZIKV infection, improved insights into the molecular basis for testes tropism and persistence is urgently needed to develop novel therapeutic strategies for future ZIKV outbreaks. We propose that during peak viremia, ZIKV enters the testis interstitium and subsequently infects the seminiferous epithelium, and because of the presence of specific immunosuppressive pathways such as Axl-IFNAR, SC become the target of long-term virus infection, although the role of PMC and SSC cannot be ruled out. The data presented here are significant as they systematically define the virus entry versus immunomodulatory role of Axl in ZIKV infection of SC. However, further investigations are warranted to delineate the role of Axl in ZIKV infection of other testis cell types and potential cross talk between Axl and viral RNA-sensing pathways such as TLR3 and RIG-I in human SC. Finally, our organoid data also demonstrate the utility of HTO to screen the efficacy of antiviral agents that clear ZIKV infection in a timely manner.

## MATERIALS AND METHODS

### Cells, testicular organoids, and virus.

Primary human SC and LC were obtained from iXCells Biotechnologies and ScienCell Research Laboratories, respectively. SC were cultured as described previously (6). LC were cultured using Leydig Cell Medium (catalog no. 4511; ScienCell) according to the manufacturer’s protocol. For human testicular organoid (HTO) experiments, HTO consisting of primary SC, LC, PMC, and SSC from adult (brain-dead donor) testicular tissue procured through the National Disease Research Interchange (NDRI) were cultured in ultralow-attachment 96-well round-bottom plates as described previously (38, 39). For seminiferous tubule mixed cell (STC) culture, seminiferous tubules isolated from human testis were digested to isolate mixed cell populations of SC, PMC, and SSC as described previously (31). STC grown on coverslips with or without vigorous washing with phosphate-buffered saline (PBS), to remove loosely attached SSC and enrich for PMC-SC, were infected with ZIKV at an MOI of 1 and then fixed using 4% paraformaldehyde (PFA) at 48 h postinfection. ZIKV strain PRVABC59 (Human/2015/Puerto Rico) was propagated once in Vero E6 cells for virus stock preparation.

### ZIKV infection and antibody/drug treatment.

Cells cultured in 96-, 24-, or 6-well plates were infected with ZIKV at different MOIs for 1 h at 37°C. The HTO were infected at 10^5^ PFU as described previously (38). For Axl receptor blockade, SC were preincubated with 10 μg/ml anti-Axl antibody (catalog no. AF154; R&D Systems) or 50 μg/ml anti-Gas6 antibody (catalog no. AB885; R&D Systems), or goat polyclonal IgG control (catalog no. AB-108-C; R&D Systems) for 2 h at 37°C prior to infection. Following infection, wells were replenished with fresh media without antibody. For Gas6 depletion, SC were serum deprived for 3 h prior to infection, infected without serum or in the presence of 5 nM recombinant human Gas6 (rhGas6) (catalog no. 885-GSB; R&D Systems), and then replenished with media containing 5% fetal bovine serum (FBS) following 1-h infection. For Axl kinase inhibition experiments, SC or HTO were preincubated with 1 μM R428 (catalog no. BGB324; Selleckchem) for 2 h prior to infection and after infection replenished with fresh media containing 1 μM R428 or DMSO vehicle control (1:10,000 dilution). ZIKV titers in the culture supernatant and intracellular ZIKV RNA was quantified by plaque assay and quantitative reverse transcription-PCR (qRT-PCR), respectively, as reported previously (48, 49).

### Immunofluorescence assay.

Cells grown on glass coverslips and fixed in 4% PFA at 48 h after ZIKV infection (MOI of 1) were permeabilized with 0.1% Triton X-100 in PBS and blocked with 5% bovine serum albumin in PBS. Cells were then incubated with primary antibodies, followed by fluorophore-conjugated secondary antibodies, and examined using an Axiocam MR camera mounted on a Zeiss Axiovert 200 microscope (48). ZIKV infection of SC, LC, and SSC was evaluated using mouse anti-ZIKV-E monoclonal antibody (1:250 dilution) produced at the Kapi’olani Community College Monoclonal Antibody Service Facility and Training Center (Honolulu, HI) using recombinant ZIKV-E produced as described previously (50) or rabbit anti-ZIKV-E (catalog no. GTX133314; GenTex) (1:500 dilution). SSC were stained for the well-established undifferentiated spermatogonia marker, ubiquitin carboxyl-terminal esterase L1 (UCHL1) (39), using primary rabbit anti-human UCHL1 (catalog no. HPA005993; Sigma) (1:1,000 dilution). Immunostaining for alpha smooth muscle actin (α-SMA; ACTA2) using primary mouse anti-human ACTA2 (catalog no. A5228; Sigma) (1:400 dilution) was used as a marker for PMC (39). Axl and IFIT1 were detected using primary rabbit anti-human Axl (catalog no. 8661; Cell Signaling) and IFIT1 (catalog no. Ab111821; Abcam) (1:250 dilution). The secondary antibodies used were Alexa Fluor 488-conjugated sheep anti-mouse and Alexa Fluor 594-conjugated goat anti-rabbit (Invitrogen; 1:500 dilution).

### Analysis of host response.

Total RNA was extracted from mock-infected and ZIKV-infected SC, LC, and HTO lysates using RNeasy mini kit (Qiagen) and synthesized into cDNA, and changes in mRNA transcripts of antiviral genes were measured by qRT-PCR, as described previously (48). The housekeeping gene *GAPDH* was used to normalize fold change values of antiviral genes, with respective mock-infected cells used as a reference control. Specific primer sequences previously reported (12) were used for *GAPDH, IFNB1*, *IFIT1*, *MxA*, *IFIH1*, and *DHX58* transcript amplification. Forward and reverse primer sequences used for other genes are shown in Table 1. Secreted IFN-β levels in SC supernatant was determined using an enzyme-linked immunosorbent assay (ELISA) kit (catalog no. DY814-05; R&D Systems) according to the manufacturer’s protocol. Protein levels of select genes in SC and LC were determined by Western blotting as described previously (51), and β-actin was used as a loading control. Specific primary antibodies used were rabbit anti-human against STAT1 (catalog no. 14994; Cell Signaling), phospho-STAT1 (catalog no. 9167; Cell Signaling), IRF3 (catalog no. 11904; Cell Signaling), phospho-IRF3 (catalog no. 29047; Cell Signaling), SOCS1 (catalog no. 3950; Cell Signaling), SOCS3 (catalog no. 2932; Cell Signaling), STAT2 (catalog no. 72604; Cell Signaling), and Axl (catalog no. 8661; Cell Signaling) and mouse anti-human β-actin (catalog no. A5316; Sigma), all at 1:1,000 dilution. Secondary antibodies (1:10,000 dilution) were conjugated with IRDye 800 and IRDye 680 (Li-Cor Biosciences), and blots were scanned using an Odyssey infrared imager.

**TABLE 1:** Primer sequences used for qRT-PCR

Gene	GenBank accession no.	Primer direction^*a*^	Primer sequence (5′–3′)
*IFNA2*	NM_000605.4	F	GAGGTTGTCAGAGCAGAA
		R	TCGTGTCATGGTCATAGC
*IFNA5*	NM_002169.2	F	CTCCTTTCTCCTGCCTGAAG
		R	AAGTGTCTCATCCCAAGTAGC
*IFNL1*	NM_172140	F	TGACACCCCACACCTTAT
		R	TCAGCCCTATGTCTCAGT
*IFNL3*	NM_001346937	F	CTGACGCTGAAGGTTCTG
		R	GCTGGGAGAGGATATGGT
*IFI6*	NM_002038	F	CAGCGTCGTCATAGGTAA
		R	CTCATCCTCCTCACTATCG
*ISG15*	NM_005101	F	AATGCGACGAACCTCTGA
		R	GCTCACTTGCTGCTTCAG
*ISG20*	NM_002201	F	CAGGGACTAGAGGCTTTC
		R	CAGTGATGGCGTAGAGAA
*IFITM3*	NM_021034	F	CTCATCGTCATCCCAGTG
		R	CGAGGAATGGAAGTTGGA

a F, forward; R, reverse.

### ZIKV luciferase reporter assay.

ZIKV containing *Nano* luciferase gene (ZIKV-Nluc), generated using strategy previously reported (45), was used to evaluate ZIKV entry into SC after Axl receptor blockade. SC, seeded in 96-well solid white microplates, were preincubated with 10 μg/ml anti-Axl (catalog no. AF154; R&D Systems), 1 μM R428, or respective goat IgG (50 μg/ml) (catalog no. AB-108-C; R&D Systems) or DMSO vehicle (1:10,000 dilution) controls for 2 h at 37°C prior to infection. SC were then infected with ZIKV-Nluc at different MOIs for 1 h in the presence of antibody or drug, washed twice with PBS, and replaced with fresh media and drug. At 4 and 24 h postinfection, the cells were washed twice with PBS, and luciferase activity (luminescence) was determined using Nano-Glo luciferase assay system (catalog no. N1150; Promega). Luciferase activity is reported as percent relative luminescence of matched time point controls.

### Cell and organoid viability assays.

SC viability was determined using the CellTiter 96 AQueous One Solution cell proliferation assay (catalog no. G3582; Promega), and HTO viability was determined by the CellTiter-Glo 3D cell viability assay (Promega G9681; Promega), according to the manufacturer’s protocols.

### Statistical analysis.

ZIKV titers, viability data, and ZIKV-Nluc luminescence data are reported as means ± standard deviations (SD) of data from ≥3 independent experiments. Gene expression data (mRNA fold change) are reported as means ± standard errors of the means (SEM) of data from ≥3 independent experiments. Statistically significant difference between data from different groups was determined by unpaired Student’s *t* test using GraphPad Prism 8.0.1 (GraphPad Software, San Diego, CA). A *P* value of <0.05 was considered statistically significant for all analyses.
